# A framework for block-wise missing data in multi-omics

**DOI:** 10.1371/journal.pone.0307482

**Published:** 2024-07-23

**Authors:** Sergi Baena-Miret, Ferran Reverter, Esteban Vegas

**Affiliations:** Departament of Genetics, Microbiology and Statistics, University of Barcelona, Barcelona Spain; Instituto Nacional de Medicina Genomica, MEXICO

## Abstract

High-throughput technologies have generated vast amounts of omic data. It is a consensus that the integration of diverse omics sources improves predictive models and biomarker discovery. However, managing multiple omics data poses challenges such as data heterogeneity, noise, high-dimensionality and missing data, especially in block-wise patterns. This study addresses the challenges of high dimensionality and block-wise missing data through a regularization and constrained-based approach. The methodology is implemented in the R package bwm for binary and continuous response variables, and applied to breast cancer and exposome multi-omics datasets, achieving strong performance even in scenarios with missing data present in all omics. In binary classification task, our proposed model achieves accuracy in the range of 86% to 92%, and F1 in the range of 68% to 79%. And, in regression task the correlation between true and predicted responses is in the range of 72% to 76%. However, there is a slight decline in performance metrics as the percentage of missing data increases. In scenarios where block-wise missing data affects multiple omics, the model performance actually surpasses that of scenarios where missing data is present in only one omics. One possible explanation for this might be that the other scenarios introduce a greater diversity of observation profiles, leading to a more robust model. Depending on the specific omics being studied, there is greater consistency in feature selection when comparing block-wise missing data scenarios.

## 1 Introduction

Recent progress in high-throughput technologies, coupled with the collaborative initiatives of international consortia like The Cancer Genome Atlas (TCGA) [1] and The Genotype-Tissue Expression (GTEx) [2], have provided access to a wealth of datasets containing diverse omic data alongside extensive clinical information for a substantial number of samples. This abundance of omic data enables a comprehensive approach to deciphering the underlying biological mechanisms of diseases and complex traits.

In the field of machine learning, it is widely acknowledged that the integration of diverse omic sources enhances the effectiveness of predictive models and facilitates more robust biomarker discovery for comprehending intricate traits [3–5]. Nevertheless, the challenges associated with managing multiple omics data are well-known, encompassing issues such as data heterogeneity, noise inherent to biological data, frequent high-dimensionality, and the presence of missing data [6].

In supervised learning, the approach to the issue of high dimensionality, aimed at eliminating irrelevant or redundant features, has been focused according to two different paradigms. The first involves incorporating regularization techniques that yield sparse models, such as Lasso or Elastic-Net [7]. Alternatively, kernel methods offer a viable strategy for circumventing the curse of dimensionality, with examples like multi-kernel learning [8]. Furthermore, hybrid approaches that combine elements of both paradigms exist, like multi-kernel learning with group-Lasso, which selects kernels on individual features to construct an optimized kernel [9].

Multi-omics studies often face an additional challenge: missing data, particularly when substantial portions of data are absent for one or more omics sources, leading to block-wise missing data. For instance, when examining sample availability in various experimental strategies within TCGA projects, a notable imbalance emerges, with RNA-seq or whole-slide imaging (WSI) samples far outnumbering those of whole-exome sequencing (WXS), whole-genome sequencing (WGS), or ATAC-seq. A similar limitation is observed in GTEx when considering the availability of RNA-seq samples across different tissues from the same donor. In both scenarios, it becomes impossible to assemble a sufficiently large dataset wherein complete information is available for each individual, encompassing all omics or all tissue types.

One potential solution involves the exclusion of samples with missing values, but this approach entails the loss of valuable data. An alternative strategy involves imputing missing data, a challenge for which numerous algorithms have been proposed [10]. Generally, these algorithms assume a random distribution of missing data, a premise that may not hold when dealing with block-wise missing data.

We encounter the two previously mentioned challenges: high dimensionality and block-wise missing data, and we have addressed them by adopting a regularization-based approach. We have retained the methodological approach outlined in [11, 12], which we have imbued it with theoretical content, ensuring a rigorous and robust formalization of every aspect, together with expanding upon to address binary prediction tasks. Additionally, we have developed the R package bwm to facilitate the implementation of this methodology for both binary and continuous response variables.

## 2 A unified feature selection model for block-wise missing multi-omic data

Consider a linear regression model involving multiple sources of data. Let ***X***^*i*^ represent a data matrix of dimensions *n* × *p*_*i*_ for the *i*-th source, *i* = 1, …, *S*, where *n* denotes the number of samples and *p*_*i*_ is the number of variables, with *p*_*i*_ ≥ 2. Additionally, let ***y*** be an *n*-dimensional vector containing the response variable values for each sample. In this model, we express the relationship as follows:

$$\begin{array}{c} y=\sum_{i=1}^{S}X^{i}\beta^{i}+\varepsilon . \end{array}$$

Here, ***ε*** represents the noise term, and $\beta^{i}\in R^{p_{i}\times 1}$ represents the set of unknown parameters for the *i*-th data source. To conduct a simultaneous analysis at both the feature and source levels, we introduce an extra parameter vector ***α*** = (*α*^1^, …, *α*^*S*^) that integrates the learned models into the regression framework:

$$\begin{array}{c} y=\sum_{i=1}^{S}\alpha^{i}\cdot X^{i}\beta^{i}+\varepsilon . \end{array}$$ (1)

Feature-level analysis is undeniably essential for extracting valuable insights from the dataset, elucidating the significance of each feature, and making informed choices regarding data preprocessing and model building. Conversely, source-level analysis empowers us to discern distinctions between various groups, pinpoint distinctive patterns or trends associated with specific sources, and make well-informed decisions grounded in these discoveries.

### 2.1 Block-missigness and profiles

In most of the cases, the data to be modeled is not complete for every data source but lack one or more data blocks. That is, matrix ***X***^*i*^ have some rows with completely missing entries, where missing rows vary among sources *i* = 1, …, *S*.

To directly apply feature-level machine learning approaches, we can discard all samples from any of the sources with missing inputs or impute missing values from the observed inputs. Both approaches have clear drawbacks. The first approach can involve a drastic reduction in the size of the data set, while the second depends largely on our prior knowledge about the underlying mechanism of missing values. Both approaches could lead to sub-optimal performances by approaching the analysis neglecting the missing block structure in the data.

When willing to preserve maximum information from the original data, one way is to partition the whole data set into multiple groups (profiles) according to the availability of data sources. In [11, 12] the notion of profile is defined from which multiple models are built with complete data.

Assuming that each observation has at least one data source available from the *S* sources in the study, then there are 2^*S*^ − 1 possible missing block patterns. We associate each observation with a profile, based on whether a certain data source is present. We establish a binary indicator vector that simplifies profiling, and which is defined as

$$I\left[1,\ldots ,S\right]=\left[I\left(1\right),\ldots ,I\left(S\right)\right]\;\;\text{where}\;\;I\left(i\right)=\{\begin{array}{cc} 1, & i\text{-th}\;\text{data}\;\text{source}\;\text{is}\;\text{available},\\ 0, & \text{otherwise}. \end{array}$$

The information contained within the indicator vector can be fully represented by a decimal integer referred, from now on, to as the *profile* (which results from converting this binary vector into a binary number and then, to a decimal number). All these profiles will be kept in a vector denoted as *pf* with a dimension of *r*. In particular, we will denote *pf* = (*m*_1_, …, *m*_*r*_).

Once the availability of data sources is known (due to the profile vector), we can break down the whole data on complete data blocks so that we can extract the maximum information of the known data. To do so, for a given profile *m*, we will group all the samples which have *m* as a profile together with those that have complete data in all the sources that are contained in the profile *m*.

**Fig 1:**
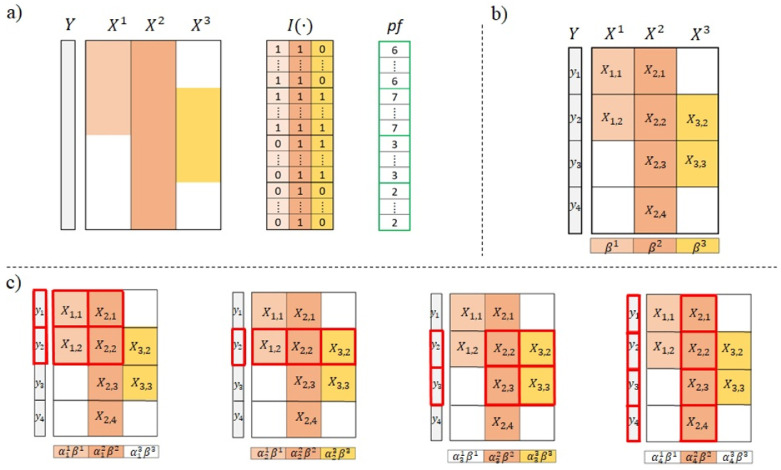
Scheme of the proposed learning model. Adapted from [12]. a) From the data matrices of multiple sources, the vector of profiles is determined. b) Crossing the profiles with the sources determines data sub-matrices. c) For each profile *m* ∈ {6, 7, 3, 2}, samples that have sub-matrices matching those in the profile, are incorporated into the regression matrix for that profile (highlighted in red).

For the sake of clarity and ease of comprehension, illustrated in Fig 1a, we have *n* samples with variables taken in three different data sources and the profile vector (once converted the profiles from binary to natural numbers) is *pf* = (6, 7, 3, 2) (so that *r* = |*pf*| = 4).

According to the profiles we can partition the ***X***^*i*^ matrices and the vector of outcomes ***y*** into boxes ***X***_*i*,*j*_ and ***y***_*j*_ respectively (see Fig 1b).

Hence, the data is divided in four groups according the availability of complete data on the sources contained on each profile, as highlighted by the red boxes Fig 1c.

Therefore, in this particular case, the goal is to learn three models ***β***^1^, ***β***^2^ and ***β***^3^ independently for each data source as well as the vectors (vectors of four components) ***α***^1^, ***α***^2^ and ***α***^3^ that combines them. Notice that, for the *i*-th data source, ***β***^*i*^ remains identical while the component $\alpha_{j}^{i}$ of ***α***^*i*^ may vary across each different profile *m*_*j*_. Further, to ease notation, we are setting to zero those components $\alpha_{j}^{i}$ that are related to missing sources instead of defining vectors ***α***^*i*^ of different dimensions; for instance, in the example of Fig 1 the parameters $\alpha_{1}^{3}$, $\alpha_{3}^{1}$, $\alpha_{4}^{1}$ and $\alpha_{4}^{3}$ are set to zero.

### 2.2 Two-stage optimization procedure

Based on (***X***^1^, …, ***X***^*S*^, ***y***), we want to estimate ***β*** = (***β***^1^, …, ***β***^*S*^) and ***α*** = (***α***^1^, …, ***α***^*S*^). To achieve this, we will apply a two-stage model. Initially, we will learn distinct models for each data source. Subsequently, we will effectively merge these learned models. In this process, we apply a regularization and constraint approach, independently at each stage, to ensure a thorough bi-level analysis. Indeed, regularization and constrained optimization are techniques used to improve model performance, prevent over-fitting, and incorporate prior knowledge into the analysis.

Let ***X***_*m*_, *m* ∈ *pf*, denote the matrix restricted to the samples that contain *m* in their profiles and *n*_*m*_ the number of rows of ***X***_*m*_. Then, $X_{m}^{i}$, *i* = 1, …, *S*, represents the *n*_*m*_ × *p*_*i*_ submatrix of the *i*-th source; that is $X_{m}=\left(X_{m}^{1},...,X_{m}^{S}\right)$, and $\alpha_{m}^{i}$ is the weight related to the matrix $X_{m}^{i}$. Moreover, let ***y***_*m*_ denote the *n*_*m*_ dimensional vector restricted to the samples that contain *m* in their profiles.

For example, in Fig 1, by taking the first profile (that is, *m* = *m*_1_ = 6) we have that

$$\begin{array}{c} X_{m}=\left(X_{m}^{1},X_{m}^{2},X_{m}^{3}\right)=(\begin{array}{ccc} \begin{array}{c} X_{1,1} \end{array} & \begin{array}{c} X_{2,1} \end{array} & \begin{array}{c} 0 \end{array}\\ \begin{array}{c} X_{1,2} \end{array} & \begin{array}{c} X_{2,2} \end{array} & \begin{array}{c} X_{3,2} \end{array} \end{array}) \end{array}$$ (2)

together with

$$\alpha_{m}=\left(\alpha_{m}^{1},\alpha_{m}^{2},\alpha_{m}^{3}\right)=\left(\alpha_{1}^{1},\alpha_{1}^{2},0\right),$$

and

$$y_{m}=(\begin{array}{c} \begin{array}{c} y_{1} \end{array}\\ \begin{array}{c} y_{2} \end{array} \end{array}).$$

We should point out here that since we will be considering the model in (1), each weight $\alpha_{m}^{i}$ will be multiplying the submatrix $X_{m}^{i}$ and, for instance, even though $X_{m}^{3}$ of the above example contains missing values, will not be taken into account since $\alpha_{m}^{3}=0$, so that the sources *i* that do not belong to a profile *m* are not acknowledged for that profile.

In [11, 12] the authors present the main ingredients needed to solve an optimization algorithm consisting on a unified feature learning model for heterogeneous block-wise missing (or even complete) data that performs both feature-level and source-level analysis simultaneously. Indeed, the problem to be solved is the following:

$$\begin{array}{c} \underset{\alpha ,\beta }{\text{min}}\frac{1}{\left|pf\right|}\sum_{m\in pf}\frac{1}{n_{m}}\varphi (\sum_{i=1}^{S}\alpha_{m}^{i}X_{m}^{i}\beta^{i},y_{m})+\lambda \Omega_{2}\left(\beta \right) \end{array}$$ (3)

subject to

$$\begin{array}{c} \Omega_{1}\left(\alpha_{m}\right)\leq 1\;\forall m\in pf, \end{array}$$ (4)

where *φ* can be any convex loss function such as, for instance, the least squares loss function or the logistic loss function. Although the least squares loss is addressed in [11, 12], here we have extended the methodology there to the logistic loss. In this way, we are broadening the scope of the earlier methodology to enable classification into two classes. This expansion is particularly relevant and valuable for practical applications.

#### 2.2.1 Computing *α* when *β* is fixed

When ***β*** is fixed, the objective function (3) is decoupled with respect to ***α***_*m*_ and, for each *m* ∈ *pf*, the optimal ***α***_*m*_ is given by the solution of the following problem:

$$\begin{array}{c} \underset{\alpha_{m}}{\text{min}}\;f\left(\alpha_{m}\right)\;\;\text{such}\;\text{that}\;\;\Omega_{1}\left(\alpha_{m}\right)\leq 1,\;\alpha_{m}=\left(\alpha_{m}^{1},\ldots ,\alpha_{m}^{S}\right)\in R^{S}, \end{array}$$ (5)

where the explicit formula of *f* depends on the loss function *φ*.

For many choices of the regularization term Ω_1_(***α***_*m*_), such as the ridge penalty, the *ℓ*_1_-norm penalty as well as other sparsity-induced penalties, the optimal solution can be efficiently computed via Ω_1_-norm projection iteration method (see Section 2 in S1 File). To do so, we first need to initialize $\alpha_{m}$, by setting each of its components to $\frac{1}{n_{m}}$, where *n*_*m*_ is the number of samples belonging to the profile *m*.

The Ω_1_-norm projection iteration procedure requires the computation of the gradient of *f* with respect to ***α***_*m*_. Below we provide the expression of such gradient for the logistic and the least squares losses.

*Logistic loss*. When $\varphi \left(z,y\right)=\sum_{l=1}^{n}\;\text{log}\;\left(1+\text{exp}\left(-y_{l}\cdot z_{l}\right)\right)$, then *f*(***α***_*m*_) is of the form

$$f\left(\alpha_{m}\right)=\sum_{l=1}^{n_{m}}\;\text{log}\;(1+\text{exp}\;[-y_{m,l}\cdot z_{m,l}])=\sum_{l=1}^{n_{m}}\;\text{log}\;(1+\text{exp}\;[-y_{m,l}\sum_{j=1}^{S}\alpha_{m}^{j}{\left(X_{m}^{j}\beta^{j}\right)}_{l}])$$

with $z_{m,l}=\sum_{j=1}^{S}\alpha_{m}^{j}{\left(X_{m}^{j}\beta^{j}\right)}_{l}$ denoting the fitted value, and where

$$X_{m}^{j}\beta^{j}={\left({\left(X_{m}^{j}\beta^{j}\right)}_{1},...,{\left(X_{m}^{j}\beta^{j}\right)}_{n_{m}}\right)}^{⊺}\in R^{n_{m}\times 1}.$$

Hence, for each *i*-th data source, the gradient ∇*f*(***α***_*m*_) with respect each $\alpha_{m}^{i}$ can be computed as follows:

$$\nabla f\left(\alpha_{m}\right)=(\partial_{1}f\left(\alpha_{m}\right),\ldots ,\partial_{S}f\left(\alpha_{m}\right))$$

with

$$\begin{array}{c} \partial_{i}f\left(\alpha_{m}\right)=-\left\langle y_{m},X_{m}^{i}\beta^{i}\right\rangle +\sum_{l=1}^{n_{m}}\frac{y_{m,l}{\left(X_{m}^{i}\beta^{i}\right)}_{l}}{1+\text{exp}\;(-y_{m,l}\cdot z_{m,l})}. \end{array}$$

*Least squares loss*. When $\varphi \left(z,y\right)=\frac{1}{2}{\left\|z-y\right\|}_{2}^{2}$, then *f* is of the form:

$$f\left(\alpha_{m}\right)=\frac{1}{2}{\left\|\sum_{j=1}^{S}\alpha_{m}^{j}X_{m}^{j}\beta^{j}-y_{m}\right\|}_{2}^{2}.$$

Further, for each *i*-th data source, the gradient ∇*f*(***α***_*m*_) with respect each $\alpha_{m}^{i}$ can be computed as follows:

$$\nabla f\left(\alpha_{m}\right)=(\partial_{1}f\left(\alpha_{m}\right),\ldots ,\partial_{S}f\left(\alpha_{m}\right))$$

with

$$\partial_{i}f\left(\alpha_{m}\right)=\alpha_{m}^{i}{\left\|X_{m}^{i}\beta^{i}\right\|}_{2}^{2}-\left\langle X_{m}^{i}\beta^{i},y_{m}\right\rangle ,$$

where 〈⋅, ⋅〉 denotes the ordinary inner product of two vectors.

#### 2.2.2 Computing *β* when *α* is fixed

When ***α*** is fixed, then (3) becomes an unconstrained regularization problem; that is,

$$\begin{array}{c} \underset{\beta }{\text{min}}\;g\left(\beta \right)+\lambda \Omega_{2}\left(\beta \right), \end{array}$$ (6)

where the explicit formula of *g* depends on the loss function *φ*. Now, we will make use of the proximal gradient iteration method (see Section 1 in S1 File). To do so, we first initialize ***β*** by learning them for each data source independently. Indeed, for that aim, both linear or Lasso regression models can be chosen. The proximal gradient iteration procedure requires the computation of the gradient of *g* with respect to ***β***. Below we provide the expression of such gradient for both the logistic and the least squares losses.

*Logistic loss*. In that case, *g*(***β***) is of the form

$$\begin{array}{cc} g(\beta ) & =\frac{1}{\left|pf\right|}\sum_{m\in pf}\frac{1}{n_{m}}\sum_{l=1}^{n_{m}}\;\text{log}\;(1+\text{exp}[-y_{m,l}\cdot z_{m,l}])\\ & =\frac{1}{\left|pf\right|}\sum_{m\in pf}\frac{1}{n_{m}}\sum_{l=1}^{n_{m}}\;\text{log}\;(1+\text{exp}[-y_{m,l}\sum_{j=1}^{S}\left(\alpha_{m}^{j}X_{m,l}^{j}\right)\beta^{j}]), \end{array}$$

where $X_{m,l}^{j}$ denotes the *l*-th row of $X_{m}^{j}$. Further, for each *i*-th data source, the gradient ∇*g*(***β***) with respect to ***β***^*i*^ can be computed as follows:

$$\nabla g\left(\beta^{i}\right)=\frac{1}{\left|pf\right|}\sum_{m\in pf}\frac{1}{n_{m}}\chi_{\left\{m\;\&\;2^{S-i}\neq 0\right\}}[-{\left(\alpha_{m}^{i}X_{m}^{i}\right)}^{⊺}y_{m}+\sum_{l=1}^{n_{m}}\frac{\alpha_{m}^{i}X_{m,l}^{i}y_{m,l}}{1+\text{exp}(-y_{m,l}\cdot z_{m,l})}],$$

where *χ*_{⋅}_ is the indicator function that returns the value 1 if the condition is met and 0 otherwise, and {*m* & 2^*S*−*i*^ ≠ 0} stands for whether the source *i* is contained (or not) on the profile *m*.

*Least squares loss*. We have

$$g\left(\beta \right)=\frac{1}{\left|pf\right|}\sum_{m\in pf}\frac{1}{2n_{m}}{\left\|\sum_{j=1}^{S}\left(\alpha_{m}^{j}X_{m}^{j}\right)\beta^{j}-y_{m}\right\|}_{2}^{2},$$

so that, for each *i*-th data source, the gradient ∇*g*(***β***) with respect to ***β***^*i*^ can be computed as follows:

$$\nabla g\left(\beta^{i}\right)=\frac{1}{\left|pf\right|}\sum_{m\in pf}\frac{1}{n_{m}}\chi_{\left\{m\;\&\;2^{S-i}\neq 0\right\}}{\left(\alpha_{m}^{i}X_{m}^{i}\right)}^{⊺}(\sum_{j=1}^{S}\alpha_{m}^{j}X_{m}^{j}\beta^{j}-y_{m}).$$

#### 2.2.3 Algorithm

The whole algorithm is shown in Algorithm 1.

**Algorithm 1** Iterative algorithm for bi-level analysis

1: **Input:** ***X***, ***y***, λ, *φ*

2: **Output:** Solutions ***α*** and ***β*** to (3)

3: Initialize ***α***_0_, see Section 2.2.1

4: Initialize ***β***_0_, see Section 2.2.2

5: **for** *t* = 1, 2, … **do**

6:  Compute ***α***^*t*^ by means norm projection iteration method, see Section 2.2.1

7:  Compute ***β***^*t*^ by means proximal gradient iteration method, see Section 2.2.2

8:  **if** the objective function on (3) stops decreasing **then**

    **return** ***α*** = ***α***^*t*^ and ***β*** = ***β***^*t*^

9:  **end if**

10: **end for**

## 3 Results

### 3.1 Breast cancer data

We used breast cancer data gathered from the source referenced in [3], which can be accessed via the link http://dx.doi.org/10.6084/m9.figshare.12052995.v1.

The dataset comprises three types of omics and clinical data obtained through the efforts of The Cancer Genome Atlas (TCGA) Research Network, accessible at https://www.cancer.gov/tcga. This dataset includes protein abundance, gene expression, and gene copy number variation data, which were employed to predict the estrogen receptor status of breast invasive carcinoma (0: negative; 1: positive). Further information on the experimental aspects of data acquisition can be found in [3] and the references therein.

The downloaded dataset contains the following components: 847 samples comprising 23410 genes with copy number variation data, 526 samples with 17815 genes featuring gene expression data, and 410 samples with 142 proteins characterized by their abundance.

In order to mitigate the challenges posed by high dimensionality in the expression data, we conducted a non-specific filtering process to exclude genes with low variance in their expression. Likewise, for copy number variation, we filtered out the less frequent variants. Subsequently, for our downstream analysis, we retained data for 2665 genes in terms of expression and 989 genes in the context of copy number variation, in addition to the abundance of 142 proteins.

**Fig 2:**
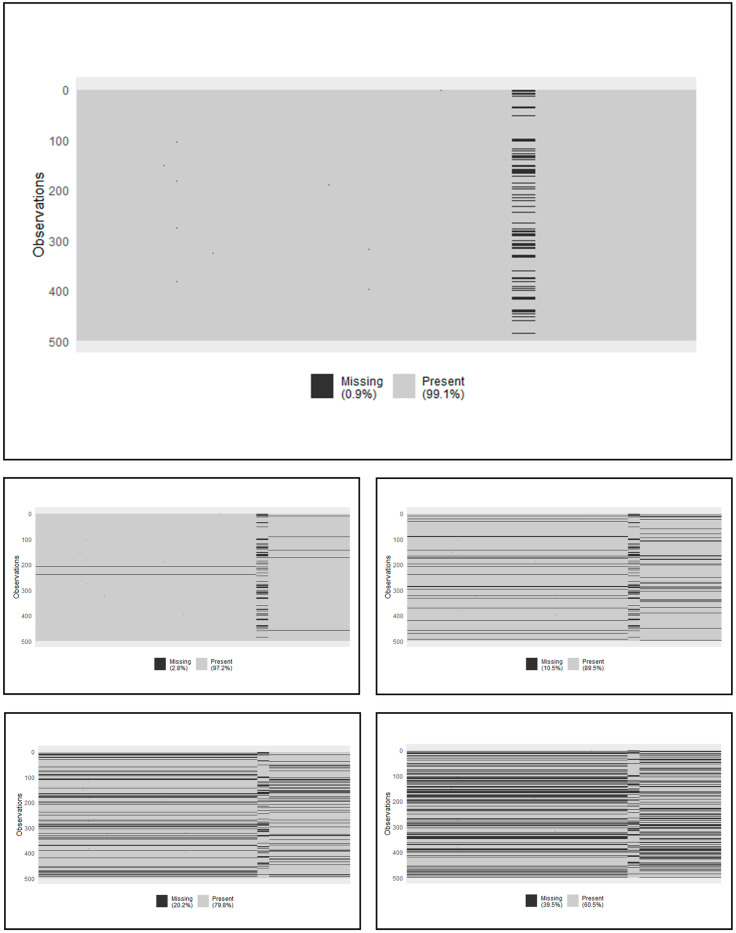
Breast cancer data: Block-wise missing scenarios. The original scenario (Top). Scenarios 2.8%, 10.5%, 20.2% and 39.5% (Bottom, from up-left to right-down).

We employed logistic loss to model the estrogen receptor status, considering its binary nature. More specifically, we focused on a subset of 499 patients, consisting of 113 with negative status and 386 with positive status. However, only 410 patients had available protein abundance data. Consequently, there were 89 patients with complete missing data in the protein abundance block, accounting for 0.9% of the overall dataset’s missingness (see Fig 2).

To further evaluate the impact of missing data blocks, we intentionally introduced random instances where certain samples had neither expression data nor copy number variation data. This ensured that the missing block structure affected all three omics. We explored several scenarios based on varying percentages of samples with missing blocks. Specifically, the scenarios examined included missing block rates of 2.8%, 10.5%, 20.2%, and 39.5%. Importantly, in these scenarios, the original level of block-missingness was retained in the protein abundance data (see Fig 2). In each of the scenarios, we divided the dataset into training (2/3) and testing (1/3) subsets. Subsequently, we trained the model on the training set and assessed its performance using the test set.

**Fig 3:**
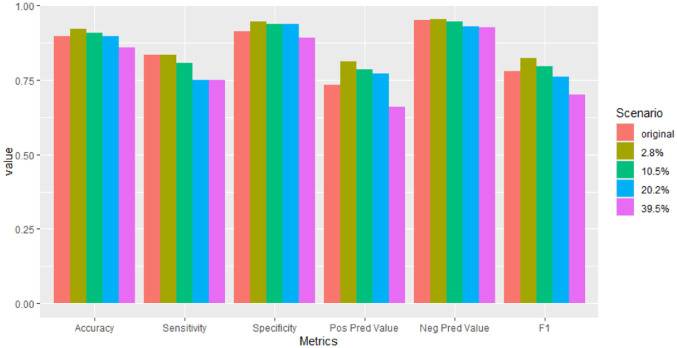
Breast cancer data: Metrics in block-wise missing scenarios.

When considering the resulting metrics on the test set (as shown in Fig 3 and Table 1), under the original scenario where block-wise missing exclusively impacts protein abundance data, our proposed methodology demonstrates strong performance. The robustness of these results was confirmed by estimating the metrics using 3-fold cross-validation (see S1 Table in S2 File).

**Table 1:** Breast cancer data: Metrics in block-wise missing scenarios.

Scenarios	Accuracy	Sensitivity	Specificity	Pos Pred Value	Neg Pred Value	F1
original	0.896	0.833	0.913	0.732	0.951	0.7792
2.8%	0.920	0.833	0.945	0.811	0.952	0.8219
10.5%	0.908	0.806	0.937	0.784	0.944	0.7945
20.2%	0.896	0.750	0.937	0.771	0.930	0.7605
39.5%	0.859	0.750	0.889	0.658	0.926	0.7013

**Fig 4:**
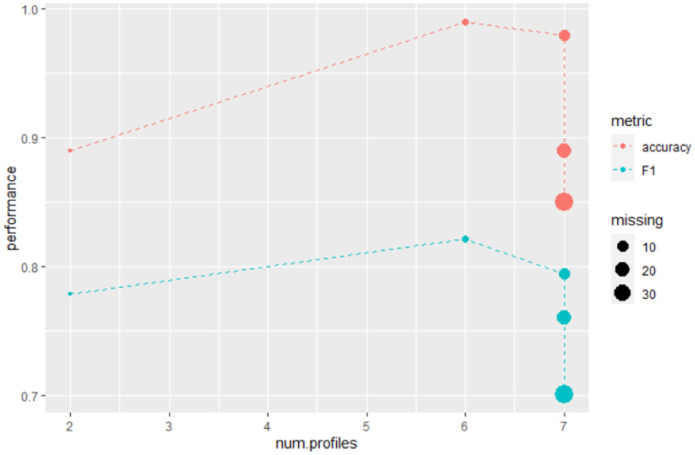
Breast cancer data: Accuracy and F1 versus profiles amount and percentage of overall missing.

In scenarios where block-wise missing data affects all omics, the metrics achieved on the test set remain notably strong. Generally, there is a slight degradation in metrics as the percentage of missing data increases. However, it is interesting to note that in some scenarios where block-wise missing affects all omics, the model performance for certain metrics surpasses that of the original scenario with missing data in only one omics (see Fig 4). One plausible explanation might be that the other scenarios introduce a greater diversity of observations profiles, with up to seven profiles, which potentially leads to a more robust model. However, when the amount of missing data is very high, the improvement provided by the number of different profiles cannot compensate for the lack of data.

To evaluate the consistency of feature selection across different block-wise missing scenarios, we determined the number of selected features within each omic that were common across these scenarios. In Table 2, moving from left to right, the boxes represent gene expression, protein abundance and gene copy number data. The diagonal of each box displays the number of selected features, while the lower triangles indicate the number of features shared between the specified scenarios. The upper triangle contains Jaccard indices, which quantify the extent of overlap among these scenarios.

**Table 2:** Breast cancer data: Feature selection consistency. From left to right, gene expression, protein abundance and gene copy number.

	orig.	2.8%	10.5%	20.2%	39.5%	orig.	2.8%	10.5%	20.2%	39.5%	orig.	2.8%	10.5%	20.2%	39.5%
orig.	495	50.9	50.7	41.5	29	123	86.9	84.3	83.2	82.4	811	81.3	78.8	61.7	74
2.8%	396	679	72.8	54.5	36.8	119	133	90.1	86.4	84.3	769	904	73	66.2	76.1
10.5%	384	558	646	59.7	41	118	127	135	89.2	91.2	666	677	700	62.9	67.2
20.2%	356	493	510	718	40	114	121	124	128	88.8	571	633	535	685	60.1
39.5%	201	290	304	319	399	112	118	124	119	125	678	729	596	551	783

We notice that there is a preference for retaining variables when missing data impacts all three omics, resulting in less sparse solutions. It can be inferred that in the absence of observations there is a tendency to preserve variables, as long as the percentage of missing data is not very high.

The extent of variable sharing is quantified using the Jaccard index. In the case of proteins and copy number data, regardless of the scenarios being compared, the Jaccard values are consistently high. However, in the domain of gene expression, the sharing of features between scenarios appears to be weaker. This difference may be attributed to the larger number of variables present in gene expression data, which comprises 2665 genes, in contrast to the other omics.

### 3.2 Exposome data

This data presented here is part of the project within ISGlobal Exposome 2021 Data Challenge framework [13], available at the same webpage of the Data Challenge, that can be found here: https://github.com/isglobal-exposomeHub/ExposomeDataChallenge2021/.

The dataset utilized is the exposome data with missing values, obtained from the exposomeNA dataset on the previous GitHub page. It consists of 1301 samples and includes 235 numeric and categorical variables. In this study only the numeric variables were chosen, retaining 1301 samples and 176 numeric variables. These variables were categorized into five blocks with different features number: Covariates (7), Exposure to chemicals (94), Indoor air (5), Lifestyles (6), and Outdoor exposures (64). The target variable is the *z-score body mass index at 6–11 years*, standardized by sex and age, using mean square error loss in the regression model. In summary, our model comprises 176 variables distributed across 5 blocks.

**Fig 5:**
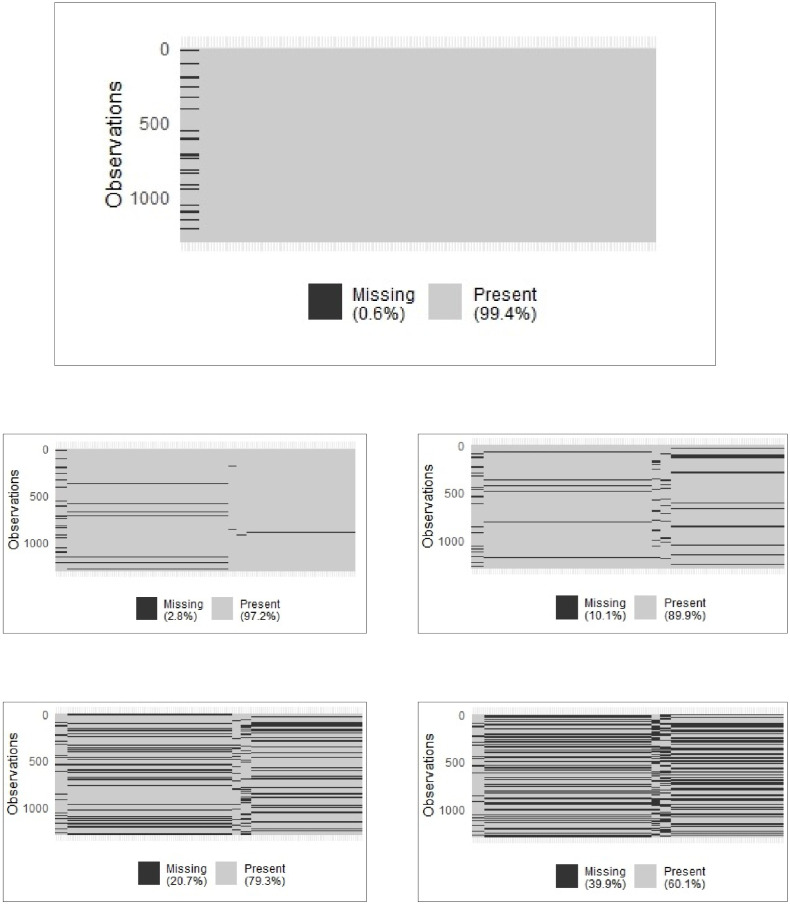
Exposome data: Block-wise missing scenarios. The original scenario (Top). Scenarios 2.8%, 10.1%, 20.7% and 39.9% (Bottom, from up-left to right-down).

In the original dataset, missing values were only present within the Covariates block, affecting 181 samples in different variables. To adapt to the missing block scenario it was necessary to discard all values in that samples, accounting for 0.6% of the missing values in the overall dataset (see Fig 5).

Similar to the breast cancer example, we assessed the impact of increasing missing blocks by introducing random missing values in the Exposure to chemicals, Indoor air, Lifestyles and Outdoor exposures blocks. Four additional scenarios were created, representing percentages of missing blocks at 2.8%, 10.1%, 20.7%, and 39.9%. It is important to note that the data from the Covariates block remained unchanged in all scenarios (see Fig 5). The dataset was then divided into training (2/3) and test (1/3) sets. For each scenario, the model was trained using the training data and its performance was evaluated using the test data.

**Fig 6:**
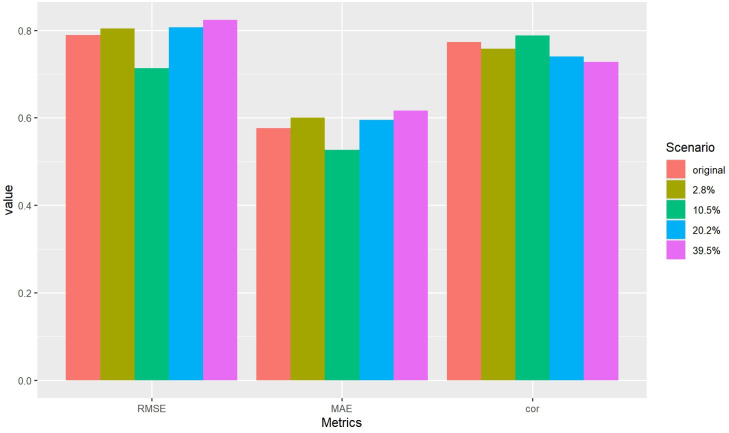
Exposome data: Metrics in block-wise missing scenarios.

**Fig 7:**
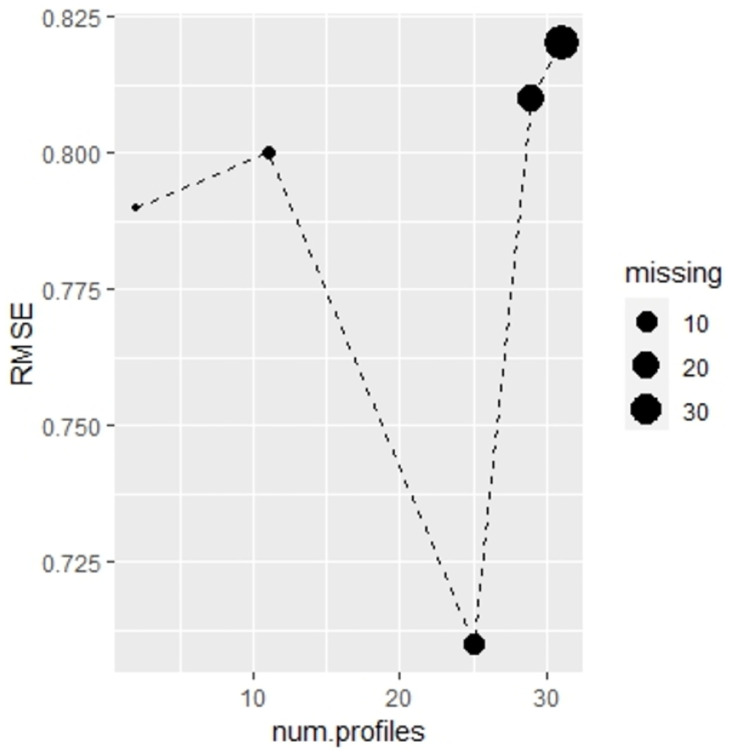
Exposome data: Accuracy versus profiles amount and percentage of overall missing.

Table 3 and Fig 6 show performance metrics of the results obtained with the test data. The metrics chosen were the root mean square error (RMSE), the mean absolute error (MAE) and the Pearson’s correlation (correlation) between true and predicted response. In the original scenario where only values are missing in the Covariates block, the performance is competitive in all metrics. However, in the four other scenarios where missing values are present in all blocks, the performance slightly decreases as the percentage of missing values increases. The robustness of these results was confirmed by estimating the metrics using 3-fold cross-validation (see S2 Table in S2 File). Nonetheless, the model still maintains a solid performance overall. Interestingly, similar to the results we have seen in breast cancer example, there is a scenario with missing values in all blocks that performs slightly better than the original scenario where only one block has missing data. Specifically, the 10.1% scenario exhibits improved performance. This behavior could possibly be attributed to a balance between a moderate number of missing values and a diverse range of observation profiles, which favor the development of a more robust model (see Fig 7).

**Table 3:** Exposome data: Metrics in block-wise missing scenarios.

Scenarios	RMSE	MAE	Correlation
original	0.789	0.576	0.773
2.8%	0.805	0.600	0.758
10.1%	0.714	0.526	0.788
20.7%	0.807	0.595	0.740
39.9%	0.824	0.616	0.728

**Fig 8:**
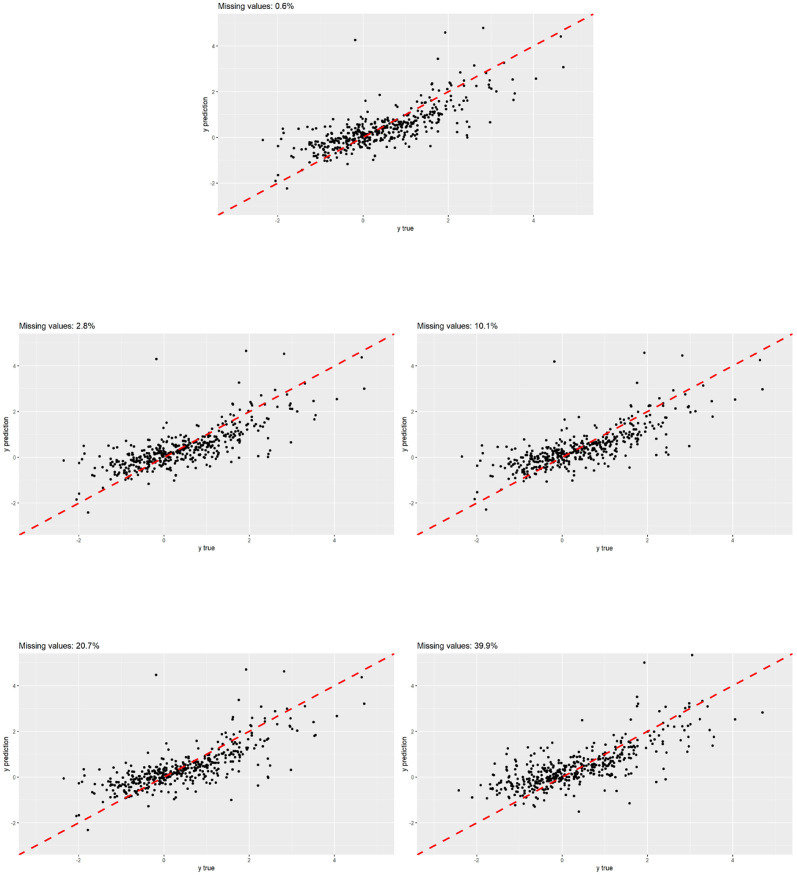
Exposome data: Scatter plots of predict values versus true values in different block-wise missing scenarios.

Fig 8 shows the scatter plots between the true response value and the predicted value in all scenarios. It can be seen how the scenario 10.1% fits better to the bisector. There is a greater correlation between the true values and the predictions. On the other hand, by increasing the percentage of missing values, the point cloud disperses around the bisector but always is close to it.

To evaluate the consistency of feature selection across different missing scenarios in each block, we measured the overlap of selected features. In blocks with a smaller number of variables, such as the Covariates, the Indoor air and the Lifestyle blocks, all or almost all variables were retained.

Table 4 shows the consistency of feature selection in the other two blocks: Exposure to chemicals (left) and Outdoor exposures (right). The presentation is similar to that used in Table 2. Therefore, within each block, the diagonal of the matrix represents the number of selected features, while the lower triangles display the number of features shared between specific scenarios. The upper triangle showcases the Jaccard indices.

**Table 4:** Exposome data: Feature selection consistency. Exposure to chemicals (left) and Outdoor exposures (right).

	original	2.8%	10.1%	20.7%	39.9%	orig.	2.8%	10.1%	20.7%	39.9%
original	90	92.5	87.1	87.1	94.7	58	77.8	85.9	73.0	84.1
2.8%	86	89	86.0	88.0	93.6	49	54	79.7	75.0	80.6
10.1%	81	80	84	84.6	88.3	55	51	61	75.0	91.9
20.7%	81	81	77	84	88.3	46	45	48	51	73.0
39.9%	89	88	83	83	93	53	50	57	46	58

In the Exposure to chemicals block, a slight decrease in the number of retained variables is observed along the diagonal as the percentage of missing values increases, except for the 39.9% scenario where the number of variables is greater than the original scenario. The Jaccard index consistently remains high across all scenarios, indicating a significant overlap of selected features.

The Outdoor exposures block exhibits slightly more irregular behavior compared to Exposure to chemicals block, yet still following similar trend. We observed an uptick in the number of variables selected in the 10.1% scenario. Overall, the Jaccard index values in this block tend to be slightly lower than those in the previous block.

## 4 Discussion

In this work, we have retained the methodological approach outlined in [11, 12], which we have imbued it with theoretical content, ensuring a rigorous and robust formalization of every aspect, together with expanding upon to incorporate the logistic loss. This expansion is particularly relevant and valuable for applications requiring to address two-class classification problems, these are often necessary steps to identify disease bio-markers. We provide an efficient approach to feature-level and source-level parameter estimation by alternating between regularization and constrain penalties, that we have implemented in the bwm R package (available at https://github.com/sergibaena-miret/Block-Wise-Missing). One of the aspects that can be improved in our model is the computational cost that we try to reduce in future implementations by parallelizing the analysis of the profiles. Currently, the analysis of a dataset of 200 observations and 1000 features with 7 block-wise missing profiles, takes 5 minutes. We trained our model on one desktop PC with 11th Gen Intel(R) Core(TM) i7–11700 @ 2.50GHz and 16GB.

To illustrate the power of the two-stage algorithm in omics data in addressing block-wise missing, we have employed it in two multi-omics data sets, such as Breast cancer [3] and Exposome data [13]. The Breast dataset provides us with a case study where it is necessary to use the logistic loss, where the variable to be predicted is the estrogen receptor status (negative/positive) and predictors comprises three blocs: gene expression, protein abundance and copy number variation. In contrast, the Exposome dataset is not as dimensional but the predictors are grouped into a higher number of blocks, and thus there is considerably more heterogeneity in the predictors. Moreover, in that case the response variable is continuous, and therefore, we apply the least squares loss.

The Breast cancer dataset has allowed us to assess the effectiveness of our implementation in dealing with various scenarios of block-missing data. The metrics used have shown satisfactory results in all cases. However, in situations where a high percentage of blocks were missing (around 40%), some metrics perform slightly worse. It is worth noting that when multiple blocks are affected by missing data instead of just one, more robust models are produced.

Through the analysis of the Exposome dataset, We have demonstrated the ability of our model to handle block-wise missing data in scenarios with a large number of profiles. The metrics used indicate competitive performance across all scenarios. As expected, the model’s performance slightly deteriorates as the percentage of missing values increases. Interestingly, in the scenario with 10.1% missing values, the model performs even better than in the original scenario. This may be due to the moderate increase in missing values, which leads to a larger number of profiles and a more robust fitted model. The impact of the trade-off between the total amount of missing data and the number of available profiles on the model’s performance will be further investigated in future research.

In summary, we have expanded a two-stage algorithm for handling block-missing data to address binary classification tasks. We have explicitly formulated the algorithm for regression models with continuous or binary response variables. Our implementation in the bwm R package is efficient and user-friendly.

## Supporting information

S1 File Appendix. (PDF)

S2 File Tables. (PDF)
