# Supplementary material for: A framework for block-wise missing data in multi-omics
Source: PLoS One. 2024 Jul 23;19(7):e0307482. doi: 10.1371/journal.pone.0307482 (PMC11265675; doi:10.1371/journal.pone.0307482)
Supplement: S1 File — (PDF) [file pone.0307482.s001.pdf]

Additional File 1: Appendix

A Framework for Block-wise Missing Data in Multi-omics

Sergi Baena-Miret[1], Ferran Reverter[1*], Esteban Vegas[1]

1 Departament of Genetics, Microbiology and Statistics, University of Barcelona, Barcelona. Spain.

* corresponding author: freverter@ub.edu

On this appendix we present two gradient iteration methods that are aimed to solve the regularization framework (equation 6 in the main text) and its constrained form (equation 5 in the main text), respectively.

1 Proximal gradient iteration method

A *proximal gradient iteration method* is a forward-backward splitting method specifically tailored to optimize an objective of the form (equation 6 in the main text) and can be described as follows [1, 2]: at each iteration $t = 1, 2, 3, \ldots$ the function g is linearized around the current point $\boldsymbol{\beta}^t$ (using its Taylor expansion) and a problem of the form

$$\min_{\boldsymbol{\beta}\in\mathbb{R}^p} g(\boldsymbol{\beta}^t) + \nabla g(\boldsymbol{\beta}^t)^\intercal(\boldsymbol{\beta} - \boldsymbol{\beta}^t) + \frac{L}{2}\|\boldsymbol{\beta} - \boldsymbol{\beta}^t\|_2^2 + \lambda\Omega_2(\boldsymbol{\beta}) \tag{1}$$

is solved. In (1), the quadratic term (i.e. the *error term*) called *proximal term*, keeps the update in a neighborhood of the current iterate $\boldsymbol{\beta}^t$ where g is close to its linear approximation, and $L > 0$ is a parameter which should essentially be an upper bound on the Lipschitz constant of ∇g. Besides, by means of the inner product induced by the norm $\|\cdot\|_2$, (1) can be rewritten as

$$\min_{\boldsymbol{\beta}\in\mathbb{R}^p} \frac{1}{2}\left\|\boldsymbol{\beta} - \left(\boldsymbol{\beta}^t - \frac{1}{L}\nabla g(\boldsymbol{\beta}^t)\right)\right\|_2^2 + \frac{\lambda}{L}\Omega_2(\boldsymbol{\beta}). \tag{2}$$

Then, a basic proximal gradient iteration method uses the solution of problem (2) as the next update $\boldsymbol{\beta}^{t+1}$. Indeed, this solution is given in terms of the *proximal operator*

$$\boldsymbol{\beta}^{t+1} := \text{Prox}_{\frac{\lambda}{L}\Omega_2}\left(\boldsymbol{\beta}^t - \frac{1}{L}\nabla g(\boldsymbol{\beta}^t)\right),$$

(we refer the reader to [3] for more details on these operators). However, in order to find such a solution is important to compute previously a suitable value for L. Often, an upper bound on the Lipschitz constant of g is not known, and even if it is, it is often better to obtain a local estimate. For instance, a suitable value for L can be obtained by iteratively increasing L by a constant factor until the condition

$$g(\boldsymbol{\beta}_L^*) \leq g(\boldsymbol{\beta}^t) + \nabla g(\boldsymbol{\beta}^t)^\intercal(\boldsymbol{\beta}_L^* - \boldsymbol{\beta}^t) + \frac{L}{2}\|\boldsymbol{\beta}_L^* - \boldsymbol{\beta}^t\|_2^2$$

is met (see [4]) where $\boldsymbol{\beta}_L^*$ denotes the solution of (2).

2 Norm projection iteration method

A *norm projection iteration method* is a forward-backward splitting method aimed to solve an objective of the form (equation 5 in the main text) whenever Ω_1 is a norm [2, 5–8]. In particular, similar as in (2), the problem (equation 5 in the main text) reduces to the projection onto the Ω_1-ball

$$\min_{\boldsymbol{\alpha}_m\in\mathbb{R}^S} \frac{1}{2}\left\|\boldsymbol{\alpha}_m - \left(\boldsymbol{\alpha}_m^t - \frac{1}{L}\nabla f(\boldsymbol{\alpha}_m^t)\right)\right\|_2^2 \qquad \text{subject to} \qquad \Omega_1(\boldsymbol{\alpha}_m) \leq \lambda,$$

and, therefore, the problem that we have to confront is: given $\hat{\boldsymbol{\alpha}}_m \in \mathbb{R}^S$, compute

$$\min_{\boldsymbol{\alpha}_m\in\mathbb{R}^S} \frac{1}{2}\|\boldsymbol{\alpha}_m - \hat{\boldsymbol{\alpha}}_m\|_2^2 \qquad \text{subject to} \qquad \Omega_1(\boldsymbol{\alpha}_m) \leq \lambda. \tag{3}$$

Now, in (3), ignoring the case $\Omega_1(\hat{\boldsymbol{\alpha}}_m) \leq \lambda$ (which has the trivial solution $\boldsymbol{\alpha}_m = \hat{\boldsymbol{\alpha}}_m$) there exists for each $\lambda > 0$ a $\mu = \mu(\lambda) > 0$ satisfying

$$\Omega_1(\text{Prox}_{\mu\Omega_1}(\hat{\boldsymbol{\alpha}}_m)) = \lambda \tag{4}$$

such that the optimization problem

$$\min_{\boldsymbol{\alpha}_m\in\mathbb{R}^S} \frac{1}{2}\|\boldsymbol{\alpha}_m - \hat{\boldsymbol{\alpha}}_m\|_2^2 + \mu\Omega_1(\boldsymbol{\alpha}_m) \tag{5}$$

has the same solution as (3). Indeed, the proximal operator $\text{Prox}_{\mu\Omega_1}(\hat{\boldsymbol{\alpha}}_m)$ is a solution of (5). Hence, if we denote $\boldsymbol{\alpha}_m^* = \text{Prox}_{\mu\Omega_1}(\hat{\boldsymbol{\alpha}}_m)$, then

$$\frac{1}{2}\left\|\boldsymbol{\alpha}_m - \hat{\boldsymbol{\alpha}}_m\right\|_2^2 + \mu\Omega_1(\boldsymbol{\alpha}_m) \geq \frac{1}{2}\left\|\boldsymbol{\alpha}_m^* - \hat{\boldsymbol{\alpha}}_m\right\|_2^2 + \mu\Omega_1(\boldsymbol{\alpha}_m^*), \qquad \forall \boldsymbol{\alpha}_m \in \mathbb{R}^S,$$

and since we are assuming that $\Omega_1(\boldsymbol{\alpha}_m^*) = \lambda$,

$$\frac{1}{2}\left\|\boldsymbol{\alpha}_m - \hat{\boldsymbol{\alpha}}_m\right\|_2^2 \geq \frac{1}{2}\left\|\boldsymbol{\alpha}_m^* - \hat{\boldsymbol{\alpha}}_m\right\|_2^2 + \mu(\Omega_1(\boldsymbol{\alpha}_m^*) - \Omega_1(\boldsymbol{\alpha}_m)) \geq \frac{1}{2}\left\|\boldsymbol{\alpha}_m^* - \hat{\boldsymbol{\alpha}}_m\right\|_2^2$$

subject to $\Omega_1(\boldsymbol{\alpha}_m) \leq \lambda$, so that $\boldsymbol{\alpha}_m^*$ is also a solution of (3). Thus, the cornerstone on solving (equation 5 in the main text) consists on finding a μ satisfying (4) and then computing

$$\boldsymbol{\alpha}_m^{t+1} = \begin{cases} \text{Prox}_{\mu\Omega_1}\left(\boldsymbol{\alpha}_m^t - \dfrac{1}{L}\nabla f(\boldsymbol{\alpha}_m^t)\right), & \text{whenever} \quad \Omega_1\left(\boldsymbol{\alpha}_m^t - \frac{1}{L}\nabla f(\boldsymbol{\alpha}_m^t)\right) > \lambda, \\ \boldsymbol{\alpha}_m^t - \dfrac{1}{L}\nabla f(\boldsymbol{\alpha}_m^t), & \text{otherwise.} \end{cases}$$

References

[1] Beck A, Teboulle M. A Fast Iterative Shrinkage-Thresholding Algorithm for Linear Inverse Problems. SIAM Journal on Imaging Sciences. 2009;2(1):183–202. doi:10.1137/080716542.

[2] Nesterov Y. Gradient methods for minimizing composite functions. Mathematical Programming. 2013;140:1436–4646.

[3] Moreau JJ. Fonctions convexes duales et points proximaux dans un espace hilbertien. Comptes rendus hebdomadaires des séances de l'Académie des sciences. 1962;255:2897–2899.

[4] Bach F, Jenatton R, Mairal J, Obozinski G. Optimization with Sparsity-Inducing Penalties. vol. 4. Foundations and Trends in Machine Learning; 2012.

[5] Balashova SD, Plaksii ZT. Projection-iteration methods for solving constrained minimization problems. J Math Sci. 1993;66:2231–2235. doi:doi.org/10.1007/BF01229589.

[6] Ceng L, Ansari Q, Yao JC. Some iterative methods for finding fixed point and for solving constrained convex minimization problems. Nonlinear Analysis: Theory, Methods & Applications. 2011;74:5286–5302. doi:10.1016/j.na.2011.05.005.

[7] Levitin ES, Polyak B. Constrained Minimization Methods. USSR Computational Mathematics and Mathematical Physics. 1966;6:1–50. doi:10.1016/0041-5553(66)90114-5.

[8] van den Berg E, Schmidt MW, Friedlander MP, Murphy KP. GROUP SPARSITY VIA LINEAR-TIME PROJECTION. UBC - Department of Computer Science. 2008;.
