# Supplementary material for: A framework for block-wise missing data in multi-omics
Source: PLoS One. 2024 Jul 23;19(7):e0307482. doi: 10.1371/journal.pone.0307482 (PMC11265675; doi:10.1371/journal.pone.0307482)
Supplement: S2 File — (PDF) [file pone.0307482.s002.pdf]

Additional File 2: Tables

A Framework for Block-wise Missing Data in Multi-omics

Sergi Baena-Miret[1], Ferran Reverter[1*], Esteban Vegas[1]

1 Departament of Genetics, Microbiology and Statistics, University of Barcelona, Barcelona. Spain.

* corresponding author: freverter@ub.edu

Table S1. Breast cancer data: Metrics in block-wise missing scenarios.

Scenarios	Accuracy		Sensitivity		Specificity		Pos Pred Value		Pos Neg Value		F1	
	mean	se	mean	se	mean	se	mean	se	mean	se	mean	se
original	0.908	0.005	0.808	0.023	0.939	0.016	0.792	0.056	0.943	0.012	0.706	0.033
2.8%	0.922	0.006	0.811	0.051	0.957	0.010	0.842	0.039	0.944	0.018	0.783	0.023
10.5%	0.928	0.003	0.818	0.032	0.962	0.015	0.860	0.057	0.946	0.013	0.791	0.031
20.2%	0.906	0.012	0.772	0.060	0.946	0.006	0.801	0.038	0.934	0.018	0.759	0.029
39.5%	0.852	0.015	0.776	0.036	0.874	0.017	0.640	0.064	0.931	0.010	0.677	0.046

Table S1 shows the mean and standard error (se) of the estimation of the performance metrics obtained by using the 3-fold cross-validation in Breast cancer data.

Table S2. Exposome data: Metrics in block-wise missing scenarios.

Scenarios	RMSE		MAE		Correlation	
	mean	se	mean	se	mean	se
original	0.785	0.013	0.583	0.01	0.751	0.020
2.8%	0.775	0.011	0.570	0.006	0.759	0.019
10.1%	0.777	0.010	0.570	0.006	0.753	0.018
20.7%	0.793	0.014	0.582	0.008	0.749	0.019
39.9%	0.827	0.001	0.609	0.011	0.722	0.014

Table S2 shows the mean and standard error (se) of the estimation of the performance metrics obtained by using 3-fold cross-validation in Exposome data.
